# Notable Developments for Vitamin D Amid the COVID-19 Pandemic, but Caution Warranted Overall: A Narrative Review

**DOI:** 10.3390/nu13030740

**Published:** 2021-02-26

**Authors:** Ronan Lordan

**Affiliations:** Institute for Translational Medicine and Therapeutics, Perelman School of Medicine, University of Pennsylvania, Philadelphia, PA 19104-5158, USA; ronan.lordan@pennmedicine.upenn.edu

**Keywords:** SARS-CoV-2, vitamin D, inflammation, immunonutrition, dietary supplements, nutraceuticals, misinformation, regulation

## Abstract

The severe acute respiratory syndrome coronavirus 2 (SARS-CoV-2) is a novel contagion that has infected over 113 million people worldwide. It is responsible for the coronavirus disease (COVID-19), which has cost the lives of 2.5 million people. Ergo, the global scientific community has been scrambling to repurpose or develop therapeutics to treat COVID-19. Dietary supplements and nutraceuticals are among those under consideration due to the link between nutritional status and patient outcomes. Overall, poor vitamin D status seems to be associated with an increased risk of COVID-19. Severely ill COVID-19 patients appear to be deficient or have suboptimal levels of serum 25-hydroxyvitamin D, a measure of vitamin D status. Consequently, vitamin D is now the subject of several prophylactic and therapeutic clinical trials. In this review, the general status of nutraceuticals and dietary supplements amid the pandemic is appraised, with a particular focus on vitamin D. Consumers should be aware of misinformation and unsubstantiated promises for products marketed for COVID-19 protection. However, maintaining a healthy diet and lifestyle will likely maintain health including optimum immune function that may affect patient outcomes. Those who are deficient in key nutrients such as vitamin D should consider lifestyle changes and potentially supplementation in consultation with their physician and/or registered dieticians.

## 1. Introduction

As of the 25 February 2021, SARS-CoV-2 has infected over 113 million people and has taken the lives of over 2.5 million globally. The pandemic has caused untold global public health and economic disruption that has extended far beyond the direct consequences of COVID-19. The clinical course of COVID-19 varies. Some individuals can be asymptomatic, and others can develop mild flu-like symptoms such as fever, headaches, nasal congestion, persistent dry cough, and/or gastrointestinal symptoms. Severely infected individuals can develop acute respiratory distress syndrome (ARDS), leading to breathing difficulties, a reduction in blood oxygen saturation requiring oxygen support or even mechanical ventilation [1,2]. Other severe complications can include abnormal blood coagulation, neurological issues, cardiovascular injury, and multiorgan failure, potentially leading to death [3,4,5]. Unfortunately, it seems that SARS-CoV-2 severely affects those most vulnerable in society including those that are immunocompromised, such as the elderly and those who have comorbidities [6]. While, COVID-19 has highlighted the incredible bravery of the healthcare and frontline workers, it has also highlighted the inequalities that exist in society and our healthcare sectors, where African Americans, Native Americans, and the Latino community have experienced a disproportionate number of COVID-19 infections and deaths [7].

Researchers are sprinting to discover or repurpose safe and efficacious prophylactics and therapeutics for COVID-19. The Food and Drug Administration (FDA) have approved the use of some treatments such as remdesivir, and other drugs such as baricitinib and bamlanivimab are available for emergency use authorization [8,9]. However, a cure for COVID-19 is still elusive. Therefore, we must rely on non-pharmacological preventative public health strategies such as physical distancing, increased hand hygiene, respiratory etiquette, the donning of face masks, the prevention of large gatherings, and effective testing and contact tracing. Collectively, these strategies are critical to reduce viral transmission, severe illness, and the loss of life [10], particularly as countries strive to keep economies functioning and education ongoing [11,12]. 

However, people have sought additional protections via the consumption of various nutraceuticals and dietary supplements [13]. Nutraceuticals are defined as supplements that are beneficial for health beyond their basic nutritional value via the prophylaxis and/or treatment of a disorder or disease. Juxtaposed, dietary supplements are intended to increase intakes of nutrients that an individual may require due to nutritional deficiency or insufficiency [14,15,16].

The beginning of the pandemic led to a spike in sales of dietary supplements, nutraceuticals, and various products purporting “immune-boosting” effects. Indeed, in one survey, ~38% of Chinese participants consumed vitamin C, probiotics, Chinese traditional herbs or other supplements associated with “immune-boosting” claims during the first lockdown of the pandemic [17]. Overall, sales of ‘natural health products’ claiming “immune-boosting” capacity grew by 34% in New Zealand over the same period. These products included vitamin C and elderberry supplements, whose sales grew by 97% and 127%, respectively [18]. In France, at the beginning of the pandemic, 36% of people planned to buy supplements to “boost immunity” and 39% intended to purchase vitamins and minerals. However, only 16% of people actually reported consuming vitamins and minerals within the same reports [19]. Globally, India is leading the markets for the highest number of new products that claim “immune system-boosting” effects [20]. The Irish company Kerry has reported that there was a trend towards people searching online for ways to supplement their diets to promote immune health [13]. They also report that healthcare professionals and those at high risk of infection seemed to increase their intake of supplements and nutraceuticals at the start of the pandemic [13,21]. These findings are supported by a Polish study that showed dietary supplements were trending online particularly at the start of the pandemic. Additionally, the usage of supplements increased from the first to the second wave of the pandemic [22]. In the same study, 60% of participants reported that they took supplements to improve immunity, 57% to improve overall health, 56% took vitamin D or fish oil as a seasonal precaution, and 53% to fill nutrient gaps in their diet. The seemingly global interest in immune-modulating supplements and nutraceuticals has also caught the attention of many scientists for their potential prophylactic or therapeutic value, but is there any supporting evidence with relevance to COVID-19? 

The nutritional status of a patient plays a significant role in the immune function and general patient health outcomes following various infections [23,24], which has also been highlighted in relation to COVID-19 [25,26,27,28,29]. There is concern that malnutrition will contribute to the worsening pandemic by increasing chronic diseases in less affluent and poverty stricken areas due to limited availability of nutritious food to support normal immune function [30]. The effects of various nutrients including vitamins and minerals have been previously investigated in vitro, in vivo, and clinically against several pathogenic viruses including coxsackievirus, polio, influenza, and other respiratory viruses [31,32,33]. Indeed, vitamin D supplementation has been associated with potential protection against respiratory infections [34] and vitamin D deficiency may increase the susceptibility of populations to seasonal influenza epidemics [35]. Vitamin D deficiency is also a significant risk factor for HIV patients, as deficiency can negatively affect the course of HIV [36,37,38]. However, supplementation may play a protective role [39,40,41], though further study is required [42]. Consequently, researchers have focused their attention on the potential for preventing or treating COVID-19 with nutraceuticals and dietary supplements such as vitamin D [14,24,43]. 

## 2. A Synopsis of Nutraceuticals and Supplements Amid the Pandemic 

**Figure 1:**
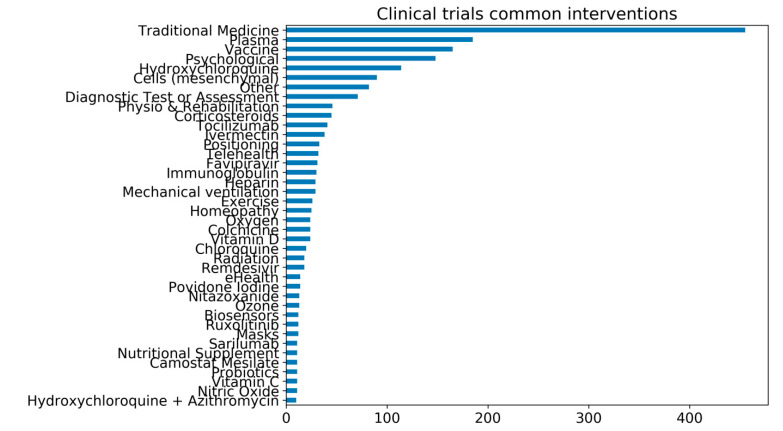
The number of common interventions under investigation for COVID-19 in clinical trials. As of 21st February 2021, there are over 6417 clinical trials registered for COVID-19. The common interventions reported in the graph are all interventions that are under investigation in at least ten clinical trials. The trial’s data are from the University of Oxford Evidence-Based Medicine Data Lab’s COVID-19 TrialsTracker [44], which has been compiled by and reproduced with permission from Rando et al. [14].

In over 6,417 clinical trials registered worldwide, vitamin D, colchicine, vitamin C, probiotics, and nutritional supplements are among some of the main interventions under investigation. Surprisingly, traditional medicines are the most common shared intervention in registered clinical trials globally (Figure 1) [14,44]. Several of the trials investigating Chinese traditional medicine have been reviewed elsewhere [45].

Vitamin C is one supplement being investigated due to its long association with optimizing immune function, reducing the length of intensive care unit (ICU) stays, and shortening the duration of mechanical ventilation for those critically ill [46,47,48,49,50,51]. However, it remains to be seen whether vitamin C can affect the clinical trajectory of COVID-19 patients, which is the subject of several clinical trials. Both healthy individuals and immunocompromised individuals should consider dietary alterations or to supplement for vitamin C if they suspect that they are deficient [14,24]. 

Zinc supplementation is also of interest and is currently under investigation, mainly as an adjuvant to other therapeutic regimes [14]. Zinc is known to exhibit antiviral and immunomodulatory properties [52,53,54]. At the beginning of the pandemic, zinc was used in combination with azithromycin and hydroxychloroquine (e.g., ClinicalTrials.gov: NCT04370782; NCT04377646), the latter is an antimalarial drug that demonstrated promising results against SARS-CoV-2 in vitro early in the pandemic [55] and was highly touted by several influential people, but ultimately failed to be of prophylactic or therapeutic value in clinical trials [56,57,58]. Adequate zinc status has been associated with antiviral immunity, less severe symptoms from common colds and flu, and lower incidence of infection [53,54,59,60,61], whereas zinc insufficiency particularly in the elderly is associated with increased infections [54]. Zinc is currently under investigation in multiple clinical trials in relation to COVID-19.

While not an exhaustive list, vitamins (A, B, E, and K), trace nutrients (selenium, copper, etc.), fiber, fish oils, probiotics, prebiotics, phenolic compounds (quercetin, resveratrol, etc.), phytomedicinal and natural products (elderberry, echinacea, rosemary, curcumin, bee propolis, etc.), melatonin, phosphatidylserine, collagen, and various other supplements and nutraceuticals are all being considered for the development of potential prophylactics and therapeutics against COVID-19 [14,21,23,24,62,63,64,65]. Nutraceuticals and supplements are also being considered for the treatment of cardiovascular-related COVID-19 symptoms. Antiplatelet agents such as Fruitflow^®^ have received some attention due to their potential to ameliorate the prothrombotic state associated with COVID-19 [66,67,68]. However, most of these agents have not been tested clinically against COVID-19. 

Since the beginning of the pandemic, there has been a growing interest in vitamin D due to immunonutritional potential, but now due to mounting evidence [43,69,70,71,72], researchers have started to give more credence to the possibility that a patient’s vitamin D status may be linked with the incidence and/or severity of COVID-19.

## 3. Vitamin D and SARS-CoV-2

### 3.1. Vitamin D

**Figure 2:**
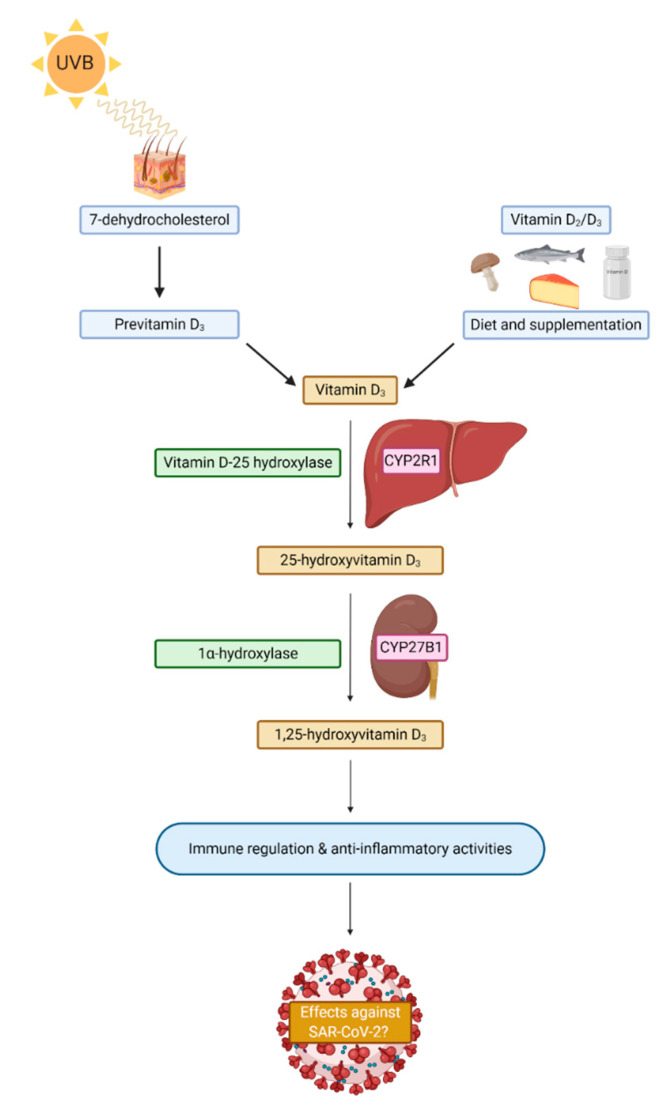
A simplistic schematic of vitamin D sources and synthesis in humans. CYP2R1 = Cryptochrome P450 familiy 2 subfamily R member 1; CYP27B1 = Crytochrom P450 family 27 subfamily B member 1; SARS-CoV-2 = Severe acute respiratory syndrome coronavirus 2.

Vitamin D is a fat-soluble steroid hormone derived from cholesterol that exists in two forms, ergocalciferol (vitamin D_2_) and cholecalciferol (vitamin D_3_), that are both endogenously produced in the dermis derived from 7-dehydrocholesterol in response to ultraviolet light (UVB) from the sun (Figure 2). Vitamin D is also obtained from dietary sources such as fish, dairy, eggs, and supplementation [24]. It is thought that 200–800 IU of vitamin D is required daily, which tends to be difficult to achieve by diet alone [72,73] but exposure to UVB light is the main contributor to the maintenance of vitamin D levels. Vitamin D circulates in the active form of 1,25-dihydroxyvitamin D [1,25(OH)2D] but this molecule only has a half-life of a few hours and tends to be present in low concentrations. Therefore, vitamin D status is estimated by measuring serum 25-hydroxyvitamin D, which is a less bioactive precursor molecule with a half-life of up to 15 days that is present in 1000-fold higher concentrations in serum [72,74]. The modern indoor lifestyle, clothing, and seasonality of daylight hours greatly impacts the level of sunlight exposure, hence why in the UK, levels of vitamin D in February are half that of the levels in September [75]. While blood concentrations of vitamin D may only last a few weeks, vitamin D stored in fat tissue can last up to 3 months [76]. There is also significant variation of 25-hydroxyvitamin D levels within populations due to physiological and environmental factors [77]. Factors such as pollution, darker skin tone, age, and pre-existing comorbidities such as obesity can also increase one’s risk of vitamin D deficiency [78,79]. Indeed, dietary intake of vitamin D may also vary due to the form consumed, as cholecalciferol is thought to be more efficacious than ergocalciferol according to nutritional trials [80,81], but that is still debated.

Vitamin D interacts with its receptor (VDR), which is present in most cell types in the body. While vitamin D is mostly known for its effects on phosphate and calcium metabolism, muscle strength, and bone mineralization [82], it is known to play a significant role in the immune system as an immunomodulatory hormone that activates approximately 200–500 genes of both the innate and adaptive immune system, indicating its importance as a biomolecule [83,84]. However, studies have shown that there is the potential for inter-individual differences in broad gene expression in human peripheral blood mononuclear cells (PBMCs) in response to vitamin D supplementation, indicating that some people may benefit from supplementation more than others [85,86,87]. Therefore, ongoing COVID-19 research should consider these potential inter-individual differences within proposed and ongoing trials. 

### 3.2. Vitamin D Status

Although highly debated, the measurement of serum 25-hydroxyvitamin D is the most common and accepted form of assessing vitamin D status. It is thought that serum 25-hydroxyvitamin D levels reliably reflect the free fractions of vitamin D metabolites, even though the bioavailable fraction are considered more clinically relevant [88,89,90,91]. One of the reasons for the controversy is the influence of the vitamin D-binding protein (DBP). Circulating 25-hydroxyvitamin D and 1,25-dihydroxyvitamin D are tightly and stably bound to DBP (85–90%) or albumin (10–15%), with only 0.03% free 25-hydroxyvitamin D and 0.4% free 1,25-hydroxyvitamin D present in circulation [92,93]. It is thought that only free and albumin-bound 25-hydroxyvitamin D are bioavailable to carry out their biological functions [94]. Notably, formulae used to determine the bioavailable vitamin D fraction in clinical settings are mostly derived from healthy populations and so these may be inaccurate for assessing vitamin D levels in critically ill patients [90].

DBP is a polymorphic protein that is denoted by its three phenotypes, which are responsible for a 5-fold difference in mean serum concentrations of DBP between the phenotypes. Median plasma concentrations of 25-hydroxyvitamin D are also contingent on DBP polymorphisms. Therefore, measurements of 25-hydroxyvitamin D in critically ill patients at single time points may lead to inaccurate determinations of vitamin D status [91,95]. Indeed, a recent study determined that calculated free 25-hydroxyvitamin D levels measured in critically ill patients are not decreased, thus it may lead to the underestimation of vitamin D levels and an overestimation of the number of patients who are vitamin D deficient [96]. Considering circulating DBP levels are also variable due to factors such as race, age, gender, pregnancy, health, and factors such as medications, oral contraceptives, and smoking [77,97], there are efforts to formulate methods that account for DBP concentrations and measures of free levels of vitamin D to accurately determine vitamin D status [77]. 

It is also important in the context of the pandemic to consider that viral infections such as HIV are known to affect DBP levels [77]. Critically ill patients generally exhibit decreased synthesis of DBP, renal wastage of 25-hydroxyvitamin D [91], and pathological situations can increase DBP synthesis in response to inflammatory cytokines such as IL-6 [93]. DBP levels may also decrease due to their association with low-density lipoproteins (LDL) in the critically ill [98]. Therefore, alterations to DBP levels may affect assessment of vitamin D status. It has been proposed that SARS-CoV-2 infections may also cause alterations to DBP [99]. Indeed, DBP polymorphisms are the subject of scientific debate as the rs7041 polymorphism may be correlated with COVID-19 prevalence and mortality [100], although further research is required [101].

DBP levels altered as a result of critical illnesses associate with and are affected by circulating LDL levels. Patients who are hospitalized due to COVID-19 have lower LDL, total cholesterol and high-density lipoproteins (HDL) upon admission, which seem to have declined further while in hospital in patients who have died. However, lipoprotein levels steadily improved to pre-illness levels in patients who recovered from COVID-19 [95,102]. Therefore, further research is required to investigate these associations and their link to vitamin D-binding protein and 25-hydroxyvitamin D levels. 

Notably, some healthcare providers occasionally measure 1,25-dihydroxyvitamin D in an effort to measure vitamin D status [103] and as a marker of chronic kidney disease [104]. However, this measurement is not considered very informative as vitamin D deficiency can lead to an increase in parathyroid hormone, which increases the activity of 1α hydroxylase thus promoting the conversion of bioavailable 25-hydroxyvitamin D into 1,25-dihydroxyvitamin D. Levels of 25-hydroxyvitamin D are present in nanogram/mL levels versus 1,25-dihydroxyvitamin D, which occurs in significantly lower picogram/mL concentrations. Therefore, measurements of 1,25-dihydroxyvitamin D may appear normal even in individuals with vitamin D insufficiency or deficiency due to increased parathyroid hormone and the activity of 1α hydroxylase [103].

A further debate in the field of vitamin D research is how to classify and define vitamin D deficiency and insufficiency. Generally, vitamin D insufficiency is defined as 25-hydroxyvitamin D levels below 21–29 ng/mL (50–75 nmol/L), whereas vitamin D deficiency is indicated by levels < 20 ng/mL (< 50 nmol/L). Severe vitamin D deficiency at < 10–12 ng/mL (< 25–30 nmol/L) is associated with the risk of osteomalacia in adults and rickets in children. Finally, 25–50 ng/mL (75–125 nmol/L) is indicative of sufficient vitamin D levels and is considered the normal range of 25-hydroxyvitamin D levels thought to prevent vitamin D deficiency and vitamin D toxicity [105,106,107]. However, some clinical subpopulations are more vitamin D deficient than others. It is well known that race is a contributory factor to vitamin D deficiency [108]. Indeed, of the total vitamin D-deficient population in the United States, 39% were Non-Hispanic African Americans and 12% were Mexican-Americans [109]. However, other clinical subpopulations such as those with obesity are also at greater risk of vitamin D deficiency [110], and this risk can be increased due to race [108]. Indeed, similar trends exist in the UK, where those of Asian ancestry (57% in winter/spring versus 50% in summer/autumn), Black African ancestry (39% versus 31%), mixed ancestry (37% versus 23%), and Chinese ancestry (33% and 21%) were more likely to be vitamin D deficient than those from White European ancestry (18% versus 5.9%) [111]. Notably, vitamin D deficiency affects 1 in 5 living in Africa [112]. Similar worrying trends exist in South America, the Middle East, China, India, and various other Asian countries [113]. In India alone, it is thought that as much as 490 million people are vitamin D deficient [114]. Looking at examples of other subpopulations, the Inuit and Amerindians are known to have low levels of 25-hydroxyvitamin D. However, they also have the increased capacity to convert the inactive forms of vitamin D into 1,25-dihydroxyvitamin D and these populations are thought to have receptors that bind more effectively. Therefore, applying the medical knowledge of vitamin D deficiency derived mostly from Caucasian populations may not apply to these subpopulations [115]. However, that also does not mean that all native subpopulations may be protected as Native Americans have disproportionate rates of SARS-CoV-2 infection and COVID-19 mortality [116], although their vitamin D status is unknown. In any case, it is clear that vitamin D deficiency and insufficiency is a global public health challenge and, as alluded to by others, it may be a pandemic in its own right [117]. 

Vitamin D status as measured by levels of 25-hydroxyvitamin D has a non-linear inverse relationship associated with acute respiratory tract infections [118,119]. Indeed, levels lower than 15 ng/mL (37.5 nmol/L) are associated with significant increased risk of acute respiratory infections [118]. It is likely that 25-hydroxyvitamin D levels may be an indicator of host immune system status due to increased risks associated with vitamin D status decline. Low vitamin D status is also a risk factor for many chronic conditions such as cardiovascular disease [120,121,122], diabetes [123], hypertension [124] and potentially some cancers [125,126,127]. Therefore, pre-existing conditions must be taken into account when assessing the vitamin D status of COVID-19 patients. 

### 3.3. Vitamin D and the Pandemic

At the beginning of the pandemic, it was speculated that the emergence of SARS-CoV-2 and possibly the severity of COVID-19 may be linked to a patient’s vitamin D levels [43,128,129]. Previously, observational studies have reported associations between low serum levels of 25-hydroxyvitamin D and susceptibility to acute respiratory tract infections [35,130,131]. Indeed, alterations of vitamin D status via supplementation may reduce the risk of developing respiratory tract infections [132,133,134]. 

The first cases of COVID-19 were reported in Wuhan China at the end of November 2019 prior to the onset of winter and quickly spread to many countries in the northern hemisphere. The outbreak occurred when vitamin D levels would be at their lowest due to winter, whereas in the southern hemisphere the levels of infection remained relatively low, when vitamin D levels would be at their highest [43,135]. This circumstantially led researchers to question whether there was a seasonal component to SARS-CoV-2 infections and whether vitamin D levels may play a role [136]. After adjusting for age, Rhodes et al. determined that there was a 4.4% increase in COVID-19 mortality for each degree of latitude north of 28°, indirectly linking a person’s vitamin D levels via exposure to UVB light to COVID-19 mortality [72], whose findings are in agreement with other published studies [137,138,139]. Indeed, surges of COVID-19 in autumn appear to correlate with latitude in Europe, indicating a potential role of vitamin D [139]. These findings seem to indicate that SARS-CoV-2 may become seasonal, which would be similar to other human coronaviruses (OC43, 229E, HKU1, and NL63) responsible for the common cold [140]. 

Several observational and retrospective studies to date have investigated the association between low 25-hydroxyvitamin D concentrations with SARS-CoV-2 incidence and COVID-19 incidence and mortality, some demonstrate potential associations [141,142,143,144,145,146,147,148,149,150,151,152], others dispute an association [153,154], and some do not report any difference in mortality between vitamin D-deficient or -replete patients [155]. Collectively, these studies have various confounding factors and limitations that need to be considered when interpreting the results. Further investigation by large-scale studies will be required to confirm these various associations. Despite these confounding factors, the data would suggest that vitamin D deficiency does play a role in the incidence of COVID-19. An initial meta-analysis seems to support the observations that individuals with poor vitamin D status tend to have a higher incidence of SARS-CoV-2 infection [156].

One might contest that many countries are particularly sunny and thus should not have major incidence of COVID-19 if vitamin D levels are a considerable factor. However, vitamin D deficiency is a widespread issue. For example, Iran is a Middle Eastern country with average sunshine of 9 h a day annually, but it has a high prevalence of vitamin D deficiency, particularly among the elderly [157,158]. Iran has been devastatingly hit by COVID-19 as infections total ~1.61 million cases and ~59,800 deaths to date [159]. A similar comparison can be drawn in Brazil, where 28% of the population are deficient and 45% have insufficient vitamin D levels despite being considered a sunny country [160]. Brazil has reported ~10.33 million cases and ~251,000 deaths to date [159]. Of course, these data are not inextricably linked, and correlation does not imply causation as many other factors are likely at play. For instance, in Brazil, there is a new variant of SARS-CoV-2 originating out of Manaus. The P.1 lineage, which has now been detected in at least 21 countries, is thought to have contributed to a massive exacerbation of SARS-CoV-2 transmission in Brazil potentially due to an increased risk of transmissibility as a result of alterations to the Spike protein that is required for viral entry to host cells as a result of genetic mutations [161,162]. It is unclear what role vitamin D deficiency may play in host immunity in light of these new SARS-CoV-2 variants. 

Furthermore, in western, southern, and eastern Europe, vitamin D deficiency is thought to affect 30–60% of individuals [163], but is less prevalent in northern Europe (<20%), possibly due to widespread vitamin D fortification [164]. Indeed, there is a substantial correlation between serum vitamin D concentrations and COVID-19 mortality across European countries [165]. In all examples presented, other factors such as diet, age, ethnicity, poverty, sun exposure, occupation, and political factors have also contributed to these devastating figures. However, it is plausible to suggest that vitamin D may also contribute to COVID-19 incidence when considered alongside the multiple observational studies published to date [141,142,143,144,145,146,147,148]. However, observational studies must also be interpreted cautiously, due to the potential for reverse causality, bias, or other confounding factors [166]. Likewise, vitamin D deficiency may also act as a surrogate indicator for an overall micronutrient deficiency that may play a role in patient outcomes.

It is also hypothesized that vitamin D may correlate with COVID-19 severity. A study of hospitalized SARS-CoV-2 patients in Spain showed that there were lower 25 hydroxyvitamin D levels and a higher prevalence of deficiency when compared to population-based controls but this study did not find an association between the level of vitamin D deficiency and the severity of COVID-19 [145]. A meta-analysis totaling 376 COVID-19 patients determined that vitamin D deficiency may be implicated in COVID-19 prognosis, although it is unclear at what levels of 25-hydroxyvitamin D they believe to be a prognostic indicator [167]. A retrospective cross-sectional study in China determined that low 25-hydroxyvitamin D levels were associated with increased incidence and severity of COVID-19 [168]. In contrast, a Mendelian randomization study found no evidence that vitamin D might be protective against SARS-CoV-2 infection or COVID-19 severity in people with European ancestry [169]. In light of these findings, the question is whether low vitamin D status reflects disease severity, or whether vitamin D status is just a bystander, or whether vitamin D status is a modifiable risk factor that can be cheaply and swiftly ameliorated [88]. Further research should determine whether vitamin D status could be used as a prognostic indicator of patient outcomes. Levels of 25-hydroxyvitamin D may provide insight into the overall immune status of a patient or the stage of disease when confounding factors such as baseline 25-hydrocyvitamin D levels and pre-existing conditions are accounted for. 

Various other notable studies in relation to vitamin D and COVID-19 have been conducted. A study in India has determined that the COVID-19 fatality rate is higher among patients with severe COVID-19 with low serum 25-hydroxyvitamin D (mean level 6.2 ng/mL; 97% vitamin D deficient) levels versus asymptomatic non-severe patients with higher levels of vitamin D (mean level 27.9 ng/mL; 33% vitamin D deficient) [170]. Notably, inflammatory markers such as IL-6, serum ferritin, and TNF-α were higher in those deficient in vitamin D. Habitual consumption of vitamin D seems to be preventative of infection in almost 8300 participants of the UK Biobank who had been tested for COVID-19 between March and June 2020 [171]. However, their vitamin D baseline measurements were over a decade old, thus it is not clear how indicative the analysis is of the present day despite the participants being habitual consumers. Another study in Chicago assessed whether vitamin D status prior to the pandemic was associated with a positive SARS-CoV-2 test. It was reported that the relative risk of testing positive for SARS-CoV-2 by polymerase chain reaction (PCR) was almost 1.8 times higher for patients likely deficient in vitamin D compared to patients with sufficient vitamin D status [172]. However, caution is warranted when interpreting these data as to reiterate, vitamin D insufficiency or deficiency has been linked to various risk factors and pre-existing conditions including age, obesity, ethnicity, and diabetes, which also happen to be risk factors for COVID-19 [24,72,173,174]. Notably, vitamin D supplementation might indirectly influence other risk factors such as hypertension and inflammation [175,176]. This may be of value as vitamin D deficiency seems to be linked with an inflammatory state of COVID-19 patients [177].

### 3.4. Plausible Mechanisms for Vitamin D against SARS-CoV-2

Mechanistically, it is plausible that vitamin D might affect aspects of SARS-CoV-2 infection [178] as vitamin D can regulate features of the acquired and innate immune system via the VDR [179]. Many immune cells express the VDR and express CYP27B1, the enzyme capable of converting 25-hydroxyvitamin D to into the active form of vitamin D (1,25-dihydroxyvitamin D) [78]. Indeed, vitamin D levels above 30 ng/mL are thought to influence the expression of 200–500 genes [72], many of which are related to the immune response to pathogenic stimuli. This includes the expression of genes involved in autophagy, the intracellular breakdown of pathogens, and the synthesis of antimicrobial peptides such as cathelicidins and defensins [178,180,181]. Cathelicidin (hCAP18/LL-37) levels tend to positively correlate with serum 25-hydroxyvitamin D levels ≤ 32 ng/mL in healthy individuals [182]. However, in critically ill patients with low 25-hydroxyvitamin D levels, cathelicidin expression appears to be suppressed [183], but it is unclear whether this expression is altered continually during the course of a critical illness [184]. Some research indicates that an increase in 25-hydroxyvitamin D levels in critically ill patients as a result of supplementation leads to a concomitant increase in cathelicidin levels [185]. During viral infections, alveolar epithelial cells have the capacity to convert inactive forms of vitamin D into 1,25-dihydroxyvitamin D, which can lead to an increased synthesis of cathelicidins [186]. 

Vitamin D downregulates the synthesis of pro-inflammatory cytokines in monocytes and macrophages by upregulating mitogen-activated protein kinase (MAPK) phosphatase-1 [187]. In experimental conditions, vitamin D may affect the renin–angiotensin system (RAS) by regulating angiotensin-converting enzyme 2 (ACE2) expression [188]. The ACE2 receptor is expressed by many cells including the surface of alveolar epithelial cells and it is the prime target for the SARS-CoV-2 Spike protein, which leads to viral replication in susceptible and permissive cells [189,190]. This binding may contribute to RAS dysregulation and the COVID-19 cytokine storm [191,192,193]. Engagement of the SARS-CoV-2 Spike protein with the ACE2 receptor via TMPRSS2 reduces its activity, leading to an increase in the activity of ACE1. This leads to the formation of additional angiotensin II (Ang II) causing amplified vasoconstriction contributing to the severity of COVID-19. Paradoxically, vitamin D analogues upregulate ACE2 expression in the lungs and reduce renin activity [188], which potentially increases the number of sites for viral entry. However, it also leads to the conversion of ang II to ang-(1-7), thus potentially reducing vasoconstriction and reducing severe acute lung injury in experimental conditions [188,194,195,196,197]. These experiments seem in part to be supported by the reduced risk of COVID-19 severity when patients have been prescribed ACE inhibitors and angiotensin receptor blockers as demonstrated by a prospective cohort study of 8.28 million people [198]. However, further research is required to explore these complex interactions.

Severe COVID-19 can cause a “cytokine storm”, a condition that leads to an elevation of cytokines and immune dysregulation, leading to systemic inflammation, a multitude of severe symptoms, multiorgan dysfunction, and potentially multiorgan failure if left untreated, which has been expertly reviewed in the context of COVID-19 by Fajgenbaum and June [199]. There has been considerable interest to further research the potential utilization of vitamin D to reduce the impact of the cytokine storm via its immunomodulatory capacity [200]. As alluded to previously, vitamin D can suppress the synthesis of pro-inflammatory cytokines including IL-6, IL17, and IL-21 [201]. Indeed, the VDR is expressed on various immune cells including macrophages [201]. However, much of the research to date has been speculative and much further research is required to determine whether vitamin D supplementation may have any effect on clinical outcomes relating to the cytokine storm. 

Overall, there is a paucity of laboratory data investigating the direct effects of vitamin D on host responses to SARS-CoV-2 specifically. Vitamin D deficiency in the elderly is known to contribute to low-grade inflammation [202], commonly associated with chronic diseases [203], of which age and pre-existing conditions are both risk factors for COVID-19. On the contrary, serum vitamin D sufficiency has been associated with a change from pro-inflammatory to an anti-inflammatory profile in older adults [202]. Systemic inflammation and low vitamin D levels can increase the risk of thrombotic complications [204,205], which are a feature of COVID-19 pathology [66,206,207]. It is thought that bioactive lipids such as platelet-activating factor (PAF) commonly associated with thrombotic and inflammatory complications [205,208] may be involved in these COVID-19 pathologies, which may be ameliorated by vitamin D [209], other supplements such as fish oils [24,210], and diet [203,211]. These are some of the many proposed mechanisms by which vitamin D may exert its putative protective effects against SARS-CoV-2 infection. Other potential anti-inflammatory and immunomodulatory effects of vitamin D have been well reviewed and critiqued elsewhere [43,72,84,165,197,212]. 

### 3.5. Prophylaxis: To Supplement, or Not to Supplement for Vitamin D

Now that the coronavirus pandemic has reaffirmed its grip over the United States and Europe, the question is whether people should prophylactically increase vitamin D intake via diet and/or supplementation for future anticipated waves of infection? While it is always important to maintain a healthy and sufficient vitamin D status, the answer for COVID-19 prevention specifically is not straightforward. Indeed, while the evidence is trending towards vitamin D deficiency as a potential risk factor for COVID-19, it is not yet clear whether increasing vitamin D intake is an effective strategy to prevent or lessen the effects of COVID-19 or reduce the incidence of infection. There is reasonable argument that it would be unwise not to supplement with vitamin D in high-risk groups [135,213], including the elderly, those with excess body fat, and the Black and minority ethnic groups, particularly African Americans who have a 6-fold higher COVID-19 mortality rate compared to white populations [135,174,214,215]. Many scientists and public health bodies have strengthened their guidelines to support vitamin D supplementation, but mostly for general health rather than COVID-19 specifically as they cite a lack of evidence yet to advise mass supplementation. Certainly, all agree that if a person suspects that they are vitamin D insufficient or deficient, they should actively ameliorate their situation by increasing exposure to sunlight, adopting dietary alterations, and/or supplementation in consultation with their physician. 

There have been suggestions by several countries and scientific organizations that high-dose supplementation of vitamin D should be considered as part of the treatment regime of infected patients and should be taken by healthcare workers who are at risk of infection [14,24,43]. The Académie Nationale de Médicine in France have recommended the implementation of a rapid testing regime of serum 25-hydroxyvitamin D for people over 60 years old, with the intention of identifying those most at risk and advising them to obtain a bolus dose of 50,000 to 100,000 IU vitamin D to limit respiratory complications. For those under the age of 60, it is further recommend that all SARS-CoV-2 positive patients take 800 to 1000 IU daily upon diagnosis [216]. Public Health England (PHE) has confirmed that they will review evidence surrounding vitamin D supplementation [217] and as of August 2020, PHE recommended that people should consider taking a daily 400 IU vitamin D dose if a person deems they have been indoors more than usual. However, they did not recommend vitamin D supplementation specifically in relation to reducing the risk of COVID-19 [218]. Similar recommendations have been echoed by Public Health Scotland (PHS) [219]. Furthermore, both PHE and PHS recommend vitamin D supplementation for BAME groups in light of evidence in relation to COVID-19 risk, although it has been highlighted that socioeconomic factors and access to healthcare certainly play a major contributory role [214,217]. Furthermore, it has been reported in the media that vulnerable Scottish individuals will receive free vitamin D supplements [220].

Slovenian doctors have now been advised to supplement vitamin D to patients in nursing homes and COVID-19 patients [221]. In Ireland, despite recommendations for vitamin D supplementation for the elderly and vulnerable by various Irish scientists [222,223], the Health Service Executive (HSE) only recommends daily supplementation if one has to self-isolate or is unable to go outside, but they did not initially provide any information on dosing [224]. However, similarly to PHE, the Irish Government has released new guidance recommending that people over the age of 65 should take a vitamin D supplement (~600 IU) daily for bone and muscle health with no specific mention of COVID-19 [225]. 

It should be noted that some countries prior to the pandemic already had vitamin D supplementation and dietary fortification strategies in place. For example, many of the Nordic countries fortify various foods with vitamin D [164] and in New Zealand there is a vitamin D supplementation program in place for all who reside in elderly-care residences to prevent deficiency [226]. Regardless of the pandemic, it is important that public health agencies consider similar public health initiatives in order to prevent vitamin D deficiency in those populations at most risk. However, as expertly highlighted by Benskin [212], most governments, medical organizations, and key leaders provide several reasons not to recommend vitamin D supplements for the general population in the present circumstances, which include overstated claims for vitamin D in the past, the risk of overdose is possible (but unlikely), the evidence for a link to COVID-19 is still under consideration, and the public might over rely on the belief that taking vitamin D will make them “immune” to COVID-19. Collectively, these are all valid concerns that public health agencies must consider before implementing widespread guidance. For those who choose to consume vitamin D supplement, it is important that they adhere to the recommended daily allowance (400 IU for children, 600 IU for 14–70 years old, and 800 IU for those over 70 years old in the United States). Doses that lead to 25-hydroxyvitamin D levels above 50 ng/mL (125 nmol/L) should be avoided to prevent the potential risk of overdosing causing toxicity and harm. The maximum safe upper level of intake for adults is 4000 IU/day [105]. While reports of vitamin D toxicity as a result of overdosing are rare, it is important that individuals adhere to public health guidance.

### 3.6. The Therapeutic Potential of Vitamin D

Vitamin D is also being explored as a therapeutic option for hospitalized patients, but there is limited evidence to support its administration to date. The COVIDIOL study has reported that early administration of calcifediol (25-hydroxyvitamin D; ~21,000 IU days 1–2 and ~11,000 IU days 3–7 of hospital admission) with hydroxychloroquine and azithromycin to 50 hospitalized COVID-19 patients led to a statistically significant reduction in ICU admissions and may have reduced disease severity versus treatment of hydroxychloroquine and azithromycin alone (26 control patients) [227]. However, several confounding factors must be taken into consideration including that serum 25-hydroxyvitamin D levels were not measured at any point in the trial and a whole host of other potential issues that have been highlighted by the National Institute for Healthcare and Excellence in the UK [228]. Despite these issues, independent follow-up statistical analyses seem to support the findings of this study as they demonstrated that the decreased ICU admissions were not due to imperfect blinding, uneven distribution of comorbidities or other prognostic indicators, but were due to calcifediol intervention [229]. In another randomized placebo-controlled trial, a daily high dose of cholecalciferol (60,000 IU) for 7 days resulted in a greater proportion of vitamin D-deficient patients becoming SARS-CoV-2 RNA negative with an accompanying significant decrease in fibrinogen due to supplementation [230]. It is proposed that cholecalciferol supplementation may result in enhanced viral clearance. These studies appear to be supported by other non-randomized trials [231,232,233].

In contrast, 240 hospitalized severe COVID-19 patients were randomized in a double-blind placebo-controlled trial with a one-time supplementation of 200,000 IU Vitamin D. While 25-hydroxyvitamin D levels increased, there was no reduction in the length of hospital stay or the requirement of mechanical ventilation [234]. The latter study used a bolus dose of vitamin D, in contrast to the other two studies, which provided lower doses but over several days. These studies provide the rationale for further exploration of the therapeutic potential of vitamin D in more carefully controlled larger trials [166]. However, investigators should consider several issues in the design of further studies, which are further elaborated in Section 3.7.

### 3.7. Perspectives on Vitamin D Clinical Trials and COVID-19

Several clinical trials are ongoing or recruiting patients to determine the potential prophylactic and therapeutic benefits of vitamin D supplementation within ClinicalTrials.gov [14] and other trial repositories. These trials may start to render answers approaching mid-late 2021 and beyond. One notable trial ‘The Vitamin D for COVID-19 (VIVID) Trial’ is highly anticipated to provide answers on the parallel testing of vitamin D_3_ supplementation for early treatment and post-exposure prophylaxis of COVID-19 [235]. Another interesting trial run by Queen Mary University of London, CORONAVIT, is using an open label, randomized, phase 3 trial enrolling 6200 participants in a test-to-treat approach to correct vitamin D insufficiency to determine whether prophylactic vitamin D may lead to reduced risk and/or severity of COVID-19 and related acute respiratory infections. The vitamin D-deficient intervention group will receive a daily dose of 800 or 3200 IU cholecalciferol versus a control group that will receive standard care of 400 IU daily (ClinicalTrials.gov Identifier: NCT04579640). The COVIDIOL pilot study is also due to continue to enroll over 1000 COVID-19 patients (ClinicalTrials.gov Identifier: NCT04366908). 

However, as discussed by Martineau and Forouhi [236], for hospital-based trials, it may be difficult to detect a response to vitamin D supplementation as patients will often present with a cytokine storm, thus it may be too late for the patient to benefit from any potential immunomodulatory or antiviral effects of vitamin D. Furthermore, as dexamethasone is a powerful anti-inflammatory agent and is now the standard of care for hospitalized patients, it might be difficult to determine any potential effect vitamin D may have on a patient’s outcome. Indeed, dexamethasone has been shown to increase DBP production, potentially leading to inaccurate estimations of vitamin D status in patients [93,237]. Therefore, as Martineau and Forouhi they have surmised, it is more likely that a prophylactic clinical benefit may be detectable in population-based trials [236].

Clinical trialists should consider the vitamin D status of a patient prior to an intervention as any potential measurable therapeutic effects of vitamin D are likely dependent on the patient’s vitamin D status prior to the intervention. Furthermore, lower levels of 25-hydroxyvitamin D in hospitalized patients might be indicative of COVID-19 severity or even the course of the disease as it is known that 25-hydroxyvitamin D levels decrease in critically ill patients [238]. Although recent reports have determined that unbound vitamin D is not reduced in critically ill patients, indicating that 25-hydroxyvitamin D concentration may underestimate vitamin D status [96]. Indeed, DBP levels should also be considered moving forward as COVID-19 infection and treatments may affect DBP levels, which may in turn affect estimations of patient vitamin D status. However, further research is required to understand these complex associations. 

In summary, it will take time before researchers can definitively determine the effects of vitamin D supplementation for the prevention and/or treatment of COVID-19. However, those most vulnerable to infection should consider vitamin D supplementation to prevent deficiency as many countries are amid another series of restrictions and lockdowns. It is important, that people focus on maintaining their nutritional, physical, and mental health by following a healthy diet, adequate sleep, and an active lifestyle. While no mitigation strategy is completely effective to prevent infection, it is important that people follow basic mitigation measures such as mask wearing, physical distancing, and adequate hand hygiene to prevent the spread of SARS-CoV-2.

## 4. Regulatory Issues and Misinformation during the Pandemic

Prior to the pandemic, the issues of regulation, safety, and efficacy loomed over any discussion in relation to dietary supplements and nutraceuticals, which have been expertly reviewed elsewhere [239,240,241]. The COVID-19 pandemic has further exposed these issues and highlighted incidences of grave scientific and public health concern. For example, there have been reports of customers exploited and persuaded to buy bogus prophylactic and therapeutics supplements for COVID-19. The spread of misinformation relating to dietary supplements and nutraceuticals has also occurred, contributing to an infodemic. 

The United States Food and Drug Administration (FDA) has issued several warning letters to companies to stop selling or distributing products under the pretense that they possess anti-COVID-19 effects. For example, the FDA released a statement about the unproven claims and the potential lethal dangers of oleandrin consumption for COVID-19 treatment and prevention [242], which is a herbal extract that was touted by influential people close to the former President of the United States [243]. However, other worrying cases have been reported. The FDA issued warnings to a nutraceutical company in California to cease deceptive advertising that claimed to provide a treatment for COVID-19 that would cost the customer thousands of dollars to avail of, leading to the company facing prosecution by the Federal Trade Commission [244]. Indeed, the FDA has also issued warning letters to companies attempting to capitalize from the unproven benefits of CBD oil products [245], ‘multi-virus defense’ supplements [246], liquid silver products [247,248], plant and algal-derived products (berberine, ecklonia cava, salidroside, oxymatrine, ashwagandh) [249], synthetic peptides (BPC-157) [249], bee propolis [250], and even mouth washes [251] all intended for COVID-19. Canada Health have also documented several similar incidences of misleading health product advertising relating to COVID-19 [252]. 

Vitamin D has also been the subject of misinformation during the pandemic. The FDA issued a warning letter to a company distributing and advertising products containing vitamin D that claimed to infer protection from COVID-19. A District Court in the state of Georgia, USA, placed a permanent injunction against the same company for the sale and distribution of these vitamin D containing products. Worryingly, these products also contained hordenine HCl, a food additive that is considered possibly unsafe to consume due to its cardiac-stimulating effects [253,254]. This example further highlights the need for surveillance in the supplement markets for safety and efficacy. 

As highlighted by Henrina et al., preprints have also contributed to some the misinformation issues amongst the general public in relation to vitamin D and COVID-19 [255]. They reported on a preprint that promoted the benefits of vitamin D for COVID-19 mortality in Indonesia. The findings of this preprint were shared widely in the news and on social media and was even cited several times. However, on further inspection, Henrina et al. discovered that the authors of the paper did not exist, which was confirmed by the Indonesian health officials that the authors alluded to being affiliated with in the preprint. Indeed, there were also several inconsistencies within the manuscript that were worrying from an ethical and scientific standpoint [255]. While the purpose of nefariously disseminating this preprint raises more questions than answers, the impact of this preprint was widespread. The link to this preprint no longer exists, but it is still available online via other internet sources allowing this misinformation to perpetuate online. These findings demonstrate a need for further vigilance and rapid dissemination of substantiated and credible information from trusted sources such as public health and regulatory agencies. Moreover, educating the general public and media companies on how to interpret scientific research is of the utmost importance moving forward to prevent the spread of misinformation on COVID-19 and vitamin D research. Indeed, better screening of preprints should be mandated to ensure that at the very least the authors identification is authenticated by preprint publishing platforms. Furthermore, it is the responsibility of scientists investigating the role of nutraceuticals and dietary supplements amid the pandemic to be cognizant of the potential reach of their claims and thus they should avoid hyperbole and perpetuating health claims in relation to COVID-19 without the support of clinical data.

## 5. Concluding Remarks

The sale of dietary supplements and nutraceuticals has significantly grown since the start of the pandemic because of consumer desire to protect themselves from potential infection and/or to mitigate the effects of COVID-19 infection. It is well established that a person’s nutritional status is linked to their immune function and patient outcomes in disease. Therefore, intuitively, following a healthy diet and lifestyle will help to maintain one’s nutritional status allowing for optimum immune function. It is still not known whether dietary supplements or nutraceuticals can efficaciously prophylactically or therapeutically alter patient outcomes against COVID-19, despite evidence that some supplements can affect outcomes such as incidence of upper respiratory tract infections, length of ICU stays, and time requiring ventilation in examples of other upper respiratory tract infections. Complications of using 25-hydroxyvitamin D as a surrogate marker of vitamin D aside, plausible evidence suggests that vitamin D deficiency is associated with increased incidence of SARS-CoV-2 infection. However, whether vitamin D insufficiency or deficiency is linked to COVID-19 severity remains to be determined. While there is insufficient evidence to suggest that vitamin D is a viable therapeutic treatment for SARS-CoV-2 infection currently, those who may be deficient should increase their levels via diet, lifestyle, and supplementation as it is cheap and relatively safe to consume to attain any potential prophylactic benefits, but more importantly because maintaining an optimum vitamin D status is essential for general health. Depending on the outcomes of forthcoming clinical trials, vitamin D may become an adjunct to any form of therapy due to its potential anti-inflammatory and immunomodulatory effects. There is no doubt that ongoing clinical trials will provide more evidence on the matter, but well-conducted large randomized and placebo-controlled trials are required to obtain definitive evidence.
